# Association between neutrophil/ high-density lipoprotein cholesterol ratio and BMI in three Argentinean Indigenous communities

**DOI:** 10.1016/j.bbrep.2025.102299

**Published:** 2025-10-25

**Authors:** Valeria Hirschler, Silvia Lapertosa, Luis Antonio Castaño, Gustavo Maccallini, Claudio Gonzalez

**Affiliations:** a Argentine Diabetes Society (SAD), Epidemiology, Buenos Aires, Argentina; b UNNE University of the Northeast, Corrientes, Argentina; c Hospital Universitario Cruces, UPV/EHU, Biocruces Bizkaia, CIBERDEM, CIBERER, Endocrinology, Bilbao, Spain; d Hidalgo Laboratories, Buenos Aires, Argentina; e CEMIC, Pharmacology, Buenos Aires, Argentina

**Keywords:** Neutrophils, High-Density lipoprotein cholesterol, Child, Indigenous people, BMI

## Abstract

**Background:**

New hematological parameters related to HDL-C can provide insights into inflammatory status and lipid metabolism.

**Objective:**

To determine the association between several cardiometabolic indices such as the neutrophil to HDL-C ratio (NHR), neutrophil to lymphocyte ratio (NLR), lymphocyte to HDL-C ratio (LHR), TG-HDL ratio, and TyG index with BMI in Argentinean Indigenous schoolchildren from three impoverished communities.

**Methods:**

Age, sex, body mass index (BMI), triglycerides (TG), HDL-C, and CBC were evaluated in a cross-sectional study. Additionally, NHR, NLR, LHR, the TG/HDL ratio, and the TyG index were calculated. Univariate and multivariate analyses were performed to explore the associations between these indices and BMI. A ROC curve was used to determine the most effective index for identifying high BMI.

**Results:**

306 schoolchildren (54.2 % female) aged 9.6 ± 2.8 years were evaluated. The prevalence of overweight/obesity was 17.3 % (n = 52). There were significant univariate correlations between BMI and the following variables: age (r = 0.244), HDL-C (r = -0.16), NHR (r = 0.27), TG-HDL -C(r = 0.23), and NLR (r = 0.23). The ROC curve demonstrated that NHR had a better discriminative performance for identifying high BMI (0.70; 95 % CI: 0.64–0.75; p < 0.01), outperforming all other indices, including NLR and HDL-C alone. Multiple linear regression analysis confirmed that NHR was significantly associated with BMI adjusted for age, sex, blood pressure, and location. (coeff. 0.24)

**Conclusion:**

This study found that NHR demonstrated better discriminative performance compared with other markers for identifying high body weight in Argentinean Indigenous children from impoverished communities.

## Highlights

• Novel hematological/HDL-C indices may serve as cardiometabolic markers.
• NHR accurately identified children with high body weight.
• NHR is a low-cost cardiometabolic risk marker in Indigenous children.

## 1 Introduction

Obesity is linked to cardiometabolic disease markers in children [1]. The Risk Factor Collaboration offers the latest worldwide estimates for individuals aged 5 to 19, indicating that in 2022, 9.3 % of boys and 6.9 % of girls were living with obesity—an equivalent of 159 million school-aged children [2]. Research has consistently shown that cardiometabolic markers increase with higher BMI percentiles among elementary school-aged children [1]. The increasing rates of overweight and obesity among children in the U.S. (affecting approximately 41.5 %), combined with its association with cardiometabolic markers, highlight the urgent need for screening and intervention to address cardiometabolic risk in children with high body weight [1]. Consistently, a multicenter study conducted by our group in 2019 across four regions of Argentina, involving more than 1200 schoolchildren, found that the prevalence of combined overweight and obesity was 42.4 % [3]. Furthermore, childhood BMI is as important as adult BMI in determining the risk of developing cardiometabolic diseases [4]. Several cardiometabolic indices associated with inflammation and dyslipidemia are linked to obesity, a major risk factor for cardiometabolic diseases [5]. With the continued increase in childhood obesity, early identification and intervention for cardiometabolic risk factors linked to high body weight remain crucial, particularly in vulnerable populations, such as Indigenous children from low socio-economic backgrounds.

Recent studies have increasingly focused on identifying early indicators of cardiometabolic risk in children, particularly inflammatory markers obtained from the complete blood count [5]. Elevated levels of neutrophils, monocytes, and lymphocytes—white blood cells present in circulating blood—are associated with peripheral inflammation and play a role in the development of cardiometabolic diseases [5]. In addition, lipid-related biomarkers play a crucial role in understanding cardiometabolic diseases. HDL-C facilitates the transport of excess cholesterol to the liver and reduces neutrophil activation, adhesion, and migration, thereby inhibiting inflammation [6]. Several new cardiometabolic indices associated with inflammation, dyslipidemia, and insulin resistance have been linked to obesity, a key risk factor for cardiometabolic diseases [5]. The most commonly used parameters include the neutrophil-to-HDL-cholesterol ratio (NHR), neutrophil-to-lymphocyte ratio (NLR), monocyte-to-HDL ratio (MHR), and triglyceride-to-HDL ratio (TG-HDL-C) [5]. These indices are appealing as systemic inflammatory biomarkers because they are inexpensive, easily accessible, and simple to calculate and could be particularly beneficial in resource-limited settings [7]. The NHR is increasingly recognized for its potential to offer a more comprehensive evaluation of inflammatory status and lipid metabolism, both of which are crucial in cardiometabolic diseases. An elevated NHR indicates either a high neutrophil count, a low HDL-cholesterol level, or both [5].

Recent evidence highlights the potential clinical utility of NHR in cardiovascular risk, as summarized in a narrative review on patients with coronary artery disease [5].

While combining multiple markers has shown potential for enhancing diagnostic and prognostic accuracy, the optimal combinations for early diagnosis and prognosis in low-income Indigenous schoolchildren remain to be identified. Limited studies have investigated new metabolic parameters associated with high body weight in Argentinean Indigenous children living in resource-limited environments. This study aimed to determine the association between several cardiometabolic indices such as the NHR, NLR, MHR, TG-HDL-C ratio, and TyG index with BMI in Argentinean Indigenous schoolchildren from three impoverished communities.

## 2 Methods

Cross-sectional data were collected from school children belonging to three distinct Indigenous communities between April 2023 and April 2024.

The first group comprised participants from Cobres (300 inhabitants) and Cerro Negro (100 inhabitants), situated at elevations of 3320 m and 3529 m above sea level, respectively. Two villages nestled in the Andes foothills, just a short distance apart, are located in the northwest of Salta Province, within the department of Rosario de Lerma, La Poma, Argentina. These small towns are home to Indigenous Colla communities [8]. The Indigenous Collas are of Andean origin, descending from ancient civilizations that historically inhabited the foothills of northwestern Argentina, Bolivia, and Peru. Their culture is closely connected to the Andes, with traditional practices related to agriculture, weaving, and Pachamama (Mother Earth) celebrations. Their rich cultural heritage significantly contributes to the region's diversity.

The second group comprised children from San Antonio de los Cobres, located in the department of Los Andes, Salta, at an altitude of 3775 m above sea level, approximately 130 km away from Cobres and Cerro Negro. This area is also home to Collas communities [9].

The third group consisted of Qom (also known as Toba) Indigenous children residing in Pampa del Indio, which is a town situated in the Libertador General San Martín Department, Chaco Province, Argentina. The town is located at an elevation of approximately 90 m above sea level. The Qom people originate from the lowland regions of the Gran Chaco, which extends across parts of Argentina, Paraguay, and Bolivia [10]. They maintain traditional crafts, languages, and customs passed down through generations. Pampa del Indio is a rural area, and the Toba community traditionally engaged in fishing, hunting, and agriculture. However, in recent years, due to social welfare programs, these communities have shifted away from traditional subsistence activities, relying more on government assistance. This transition has led to cultural changes, with traditional occupations being replaced by dependence on social benefits. Two schools were randomly selected from San Antonio de los Cobres and from Pampa del Indio. In Cobres and Cerro Negro, there was only one school in each location, and these schools were selected by default. All children attending these schools whose parents provided informed consent were included in the study. The consent rate was 50 %. Ninety-nine percent of the population in these regions comprises Indigenous children Collas or Tobas. The absence of intermarriage with other ethnic groups within their family histories ensures that the three groups remain ethnically homogeneous.

Exclusion criteria comprised the lack of age, sex, weight and height information, no lab measurement, the presence of diabetes or other chronic diseases, and failure to sign the informed consent form. Approval for the study was granted by the Human Rights Committee of the University of the Northeast in Corrientes (UNNE RES - 2024 - 106 -). Furthermore, both parents and subjects signed written consent after the research had been thoroughly explained.

Data collected included age, sex, blood pressure (BP), height and weight. Parents were classified into socioeconomic levels based on educational attainment. Height and weight were measured with subjects wearing light clothing and without shoes. Height was recorded to the nearest 0.1 cm with a wall-mounted stadiometer. Weight was measured to the nearest 0.1 kg on medical scales. Children were classified as underweight (<5th percentile), normal weight (5th to < 85th percentile), overweight (85th to < 95th percentile), or obese (≥95th percentile) according to CDC norms. BMI z-score (BMI-z) was also determined [11]. The waist circumference measurement was conducted at the umbilical level and recorded to the nearest 0.1 cm. A flexible, non-elastic tape measure was employed while the child stood without clothing obscuring the waist area.

Blood samples were collected from children through venipuncture. Various analyses, including a complete blood count, lipid levels, and glucose levels were conducted.

A complete blood count is a laboratory test used to measure the levels of red and white blood cells, platelets, hemoglobin, and hematocrit in an individual's blood [12].

The lipids were assessed using standardized methods, utilizing the Architect c 16000 instruments from Toshiba (Tokyo, Japan) and dedicated reagents supplied by Abbott Laboratories (Abbott Park, IL), while the glucose levels using the glucose oxidase enzymatic method (Roche Diagnostics). All samples underwent analysis at a single laboratory, and a standardized homogeneous assay was employed to analyze them. Serum samples had been stored at −70 °C, and both groups were evaluated simultaneously.

Indices such as NHR, NLR, MHR, TG-HDL ratio, and TyG index were calculated. The calculations for NHR, NLR, LHR, TG/HDL-C ratio, and TyG index were performed using the following formulas.

• Neutrophil-to-HDL-C ratio (NHR) = Neutrophil relative value (%)/ HDL-C level (mg/dL)
• Neutrophil-to-Lymphocyte ratio (NLR) = Neutrophil relative value (%)/Lymphocyte relative value (%)
• Monocyte-to-HDL-C ratio (MHR) = Monocyte relative value (%)/HDL-C level (mg/dL)

Insulin resistance indices were calculated as follows.

• TG/HDL-C ratio = Total triglycerides (mg/dL)/HDL-C level (mg/dL)
• TyG index = Ln [Fasting triglycerides (mg/dL) × Fasting plasma glucose (mg/dL)/2]. The TyG index is expressed on a logarithmic scale [13].

## 3 Data analysis

Descriptive statistics for the raw variables were reported as mean ± SD or as proportions. Chi-squared tests were employed to compare proportions between groups. The Shapiro-Wilks test was used to assess the normality of continuous variables. For comparisons between two groups with normally distributed data, a Student's t-test was conducted. When multiple comparisons were made, Bonferroni's adjustment was applied. If the assumption of homogeneity of variances was violated, the Brown–Forsythe test was utilized.

When comparing more than three groups with normally distributed data, a one-way analysis of variance (ANOVA) was performed, followed by the Student-Newman-Keuls post hoc test. In cases where homogeneity of variances could not be confirmed, the nonparametric Kruskal-Wallis test was used instead of ANOVA, with Dunnett's post hoc test applied for multiple comparisons.

To evaluate the strength of association between two variables, Pearson and Spearman correlation coefficients were calculated. The primary objective of this study was to examine the association of the NHR, NLR, LHR, TG-HDL ratio, and TyG index with BMI among Argentinean Indigenous schoolchildren from three impoverished communities. Multiple linear regression analysis was conducted with the NHR or NLR as the dependent variable, and age, sex, location, and BMI as independent variables.

The variables included as potential confounders in the multivariate analysis were those that showed significant correlations in the univariate analysis, such as BP. In addition, biological variables such as age and sex were included as adjustment factors, regardless of their univariate significance, given their role as classical biological determinants.

The Receiver Operating Characteristic (ROC) curves were utilized to identify the most effective index for detecting high body weight (defined as BMI in the upper quartile).

The CDC criteria for overweight/obesity were used only to report prevalence. For the ROC curve analysis, due to the low prevalence of overweight and obesity, “high BMI” was instead defined as the upper quartile of the BMI percentile distribution to allow sufficient statistical power. It is important to note that an area under the ROC (AUC) < 0.7 indicates poor to fair discrimination, meaning the test is not particularly strong in distinguishing between the two conditions (e.g., high vs. normal body weight). The AUCs were calculated for all the indices evaluated, and comparisons were made between these areas to identify the marker with the better discriminative performance for high body weight. Differences between AUCs were evaluated using the DeLong test for correlated ROC curves. Furthermore, the ROC curves were also used to determine the optimal cutoff points for different indices in indicating high body weight. The optimal threshold was identified as the point closest to the upper left corner of the ROC curve, where sensitivity and specificity are maximized—specifically, where their curves intersect. These cutoff values represent the levels above which there is an increased likelihood of having high body weight. All statistical analyses were performed using SPSS software (version 22.0; IBM Corp., Chicago, IL, USA).

## 4 Results

A total of 357 children were initially recruited from six elementary schools in Argentina. However, two were later excluded due to thyroid medication use, ten declined to participate, and BMI data were missing for 39 participants due to measurement errors and incomplete records. Thus, the final sample consisted of 306 children (166 girls) who were examined.

The prevalence of obesity was 3.7 % (n = 11). The prevalence of overweight and obesity was 17.3 % (n = 52). Regarding residence area, we found that the prevalence of overweight and obesity was significantly higher in Pampa del Indio (25.4 %) than in the San Antonio de los Cobres (16.5 %) and Cobres/Cerro Negro towns (11.3 %). Participants were from a low socioeconomic background, as evidenced by the educational level of their parents—76.3 % of whom had completed only elementary school or less.

### 4.1 Blood profile in the three communities

Table 1 shows blood profile results in the three communities. Hemoglobin and hematocrit were significantly higher and increased with altitude. In contrast, leukocyte count and neutrophil percentages were significantly higher in Pampa del Indio, possibly related to endemic parasitic infections (including Chagas disease), as suggested by the significantly higher eosinophil levels. At high altitude, hypoxia may influence hemoglobin levels through enhanced erythropoiesis, oxidative stress, and systemic inflammation.

**Table 1:** Blood Profile in the Three communities.

	San Antonio de los Cobres Altitude 3755 m		Pampa del Indio Altitude 93 m		Cobres Altitude 3050 m	
Hematocrit % a	48.14	±3.35	38.17	±3.26	43.39	±2.96
Hemoglobin g/dL^a^	16.05	±1.12	12.72	±1.09	14.46	±0.99
Leucocytes n/μL a	7726	±2297	9556	±3124	6672	±1387
Neutrophils % b	52.85	±10.35	54.65	±8.8	45.07	±10.35
Eosinophyls % c	3.72	±3.39	5.49	±2.01	3.13	±3.18
Lymphocytes % b	37.91	±9.07	39.9	±9.38	43.62	±9.14

Data are presented as mean ± SD. Abbreviations; San Antonio de los Cobres (SAC), Pampa del Indio (PI), and Cobres (C).Significant differences (p values < 0.01) were found between.

a SAC vs PI vs C.

b SAC & PI vs C, and.

c SAC & C vs. PI.

### 4.2 Clinical and metabolic characteristics

Mean levels of various characteristics are presented according to sex in Table 2. A total of 306 elementary school students (54.25 % female) with a mean age of 9.6 years, a mean BMI of 18 (corresponding to the 55th percentile per CDC), a mean systolic BP of 107 mmHg, a mean HDL-C level of 43 mg/dL, and a mean TG level of 119 mg/dL, were evaluated. There were no significant differences in the studied variables between sexes, except for the number of leukocytes, which was significantly higher in girls than in boys.

**Table 2:** Clinical and Metabolic characteristics according to sex.

	Males (n = 140)		Females (n = 166)		Total (n = 306)	
Age years	9.45	±2.91	9.72	±2.62	9.60	±2.76
BMI	17.98	±2.87	18	±2.9	17.99	±2.88
BMIPercent.	51.97	±28.89	57.56	±26.03	54.99	±27.48
SBP mmHg	106.9	±20.78	107.38	±22.45	107.16	±21.68
DBP mmHg	70.99	±13.29	71.08	±13.03	71.04	±13.13
Hb g/dL	14.89	±1.49	15.02	±1.82	14.96	±1.68
Leucocytes n/μL ∗	7418	±2160	8089	±2674	7798	±2482
Neutrophils %	49.69	±10.08	52.12	±11.08	51.07	±10.71
HDL-C mg/dL	42.97	±8.63	42.55	±9.74	42.74	±9.25
TG mg/dL	114.82	±59.55	121.7	±47.5	118.57	±53.31
NLR	1.39	±0.83	1.54	±1.13	1.47	±1.02
NHR	1.19	±0.34	1.28	±0.39	1.24	±0.37
MHR	0.14	±0.05	0.13	±0.05	0.13	±0.05
TyG	8.3	±0.49	8.35	±±0.38	8.33	±0.43
TG-HDL-C	2.9	±2.02	3.11	±1.74	3.01	±1,87

Data are presented as mean ± SD according to sex. CBC-derived inflammatory indices and cardiometabolic markers are depicted. The p-value indicates the comparison between the sexes. ∗A p-value = 0.027 was considered statistically significant. NHR: neutrophils-to-HDL-C ratio; MHR: monocytes-to-HDL-C ratio; NLR: neutrophils-to-lymphocytes- ratio; TyG index; TG-HDL-C ratio triglycerides-to-HDL-C ratio.

### 4.3 BMI percentile quartiles

**Fig. 1:**
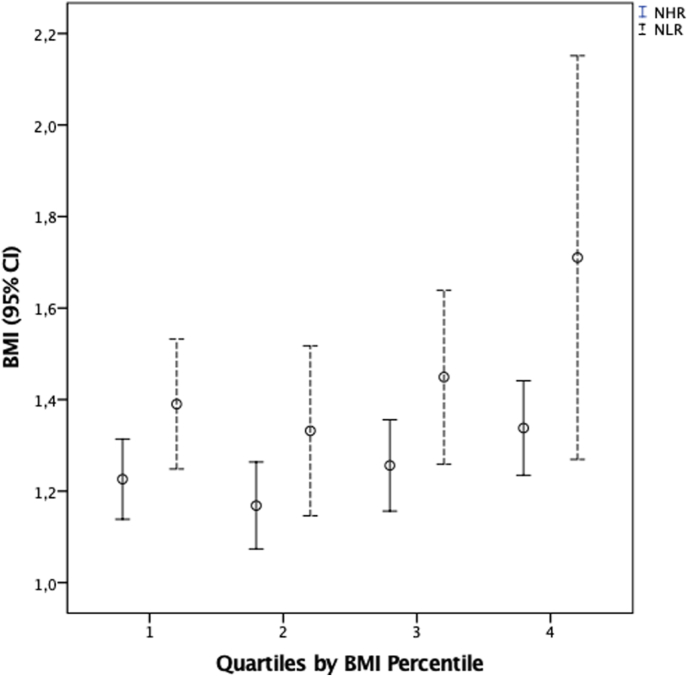
Mean children's NHR & NLR and 95 % Confidence Intervals Across BMI Percentile Quartiles. NHR in Q4 was significantly higher than in Q1–Q3 NHR: neutrophils-to-HDL-C ratio; NLR: neutrophils-to-lymphocytes- ratio.

Children were divided into four groups according to BMI percentile quartiles for comparison of their means, defined as 1st (n.77; 17 ± 10), 2nd (n.77; 46 ± 8), 3rd (n.76; 88 ± 6), and 4th (n.76; 55 ± 27) (Table 3). There were no significant differences in mean age, systolic BP, hemoglobin, HDL-C, TG-HDL-C, and TyG across the quartiles. However, diastolic BP was higher in quartile 3 compared to quartile 1. Additionally, TG, leukocytes, and neutrophils were significantly elevated in quartile 4 compared to quartile 1. Furthermore, NHR and NRL were significantly higher in quartile 4 compared to quartiles 1, 2, and 3 (Fig. 1). Conversely, MHR was significantly lower in quartile 3 compared to quartiles 1 and 4.

**Table 3:** Clinical and Metabolic characteristics by BMI percentile Quartiles.

	Quartile 1 N = 77 (BMI perc 17 ± 10)		Quartile 2 N = 77 (BMI perc 46 ± 8)		Quartile 3 N = 76 (BMI perc 68 ± 5)		Quartile 4 N = 76 (BMI perc 88 ± 6)		Total N = 306 (BMI perc Median 59 IQR: 33–78)	
Age years	9.55	±2.82	9.85	±2.86	9.67	±2.52	9.44	±2.85	9.63	±2.76
SBP mmHg	101.53	±22.12	106.36	±22.40	110.18	±21.93	110.81	±19.36	107.22	±21.70
DBP mmHg a	67.25	±12.51	70.09	±13.77	74.30	±13.31	72.62	±12.19	71.07	±13.17
Hb g/dL	14.74	±1.60	15.00	±1.63	15.21	±1.70	14.97	±1.80	14.98	±1.68
Leucocytes b	7225	±2383	7572	±2381	7854	±2021	8689	±3024	7799	±2497
Neutrophils b	48.40	±9.29	49.12	±11.08	52.73	±10.69	54.86	±10.82	51.12	±10.73
HDL-C mg/dL	43.39	±8.71	41.98	±8.50	44.84	±9.83	40.65	±9.74	42.75	±9.27
TG b mg/dL	108.42	±41.32	114.54	±42.60	117.28	±51.01	135.58	±73.15	118.45	±53.50
NLR c	1.35	±0.93	1.34	±0.70	1.50	±0.76	1.79	±1.54	1.48	±1.02
NHR^c^	1.15	±0.31	1.20	±0.32	1.23	±0.43	1.42	±0.37	1.24	±0.37
MHR^d^	0.15	±0.05	0.14	±0.05	0.11	±0.04	0.13	±0.05	0.13	±0.05
TyG	8.27	±0.44	8.28	±0.40	8.33	±0.41	8.43	±0.46	8.33	±0.43
TG-HDL-C c	2.64	±1.21	2.89	±1.39	2.88	±2.01	3.69	±2.55	3.01	±1.88

Data are presented as mean ± SD. Abbreviations; BMI body mass index; BMI perc BMI percentile, BP blood pressure, TG triglycerides, neutrophil to HDL-C ratio (NHR), neutrophil to lymphocyte ratio (NLR), monocyte to HDL-C ratio (MHR), TG-HDL ratio, and TyG index. Percentile is a quantitative measure of the deviation of a specific variable taken from the mean of that population. CDC BMI percentile takes into account age and sex.

Significant differences (p values < 0.05) were found between.

a Q3 vs Q1.

b Q4 vs Q1.

c Q4 vs Q2, Q3&Q1.

d Q3 vs Q1&Q2.

### 4.4 Univariate correlations

Significant univariate correlations were observed between BMI and the following variables: age (r = 0.244; p < 0.01), HDL-C (r = -0.16; p = 0.01), NHR (r = 0.27; p < 0.01), systolic BP (r = 0.18; p < 0.01), diastolic BP (r = 0.16; p < 0.01), TG-HDL -C(r = 0.23; p < 0.01), and NLR (r = 0.23; p < 0.01).

### 4.5 ROC curves

**Fig. 2:**
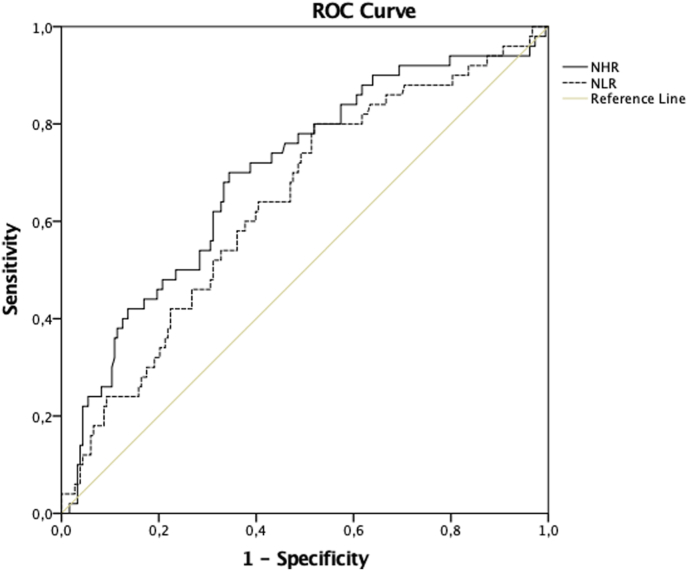
Area under the ROC curve of NHR & NLR, for predicting high body weight (BMI percentile) >3rd quartile in children. NHR: neutrophils-to-HDL-C ratio; NLR: neutrophils-to-lymphocytes- ratio.

To assess which marker which marker had better discriminative performance than others for identifying children with high body weight, ROC curves were generated for NHR, NLR, MHR, TyG, TG-HDL-C, and HDL-C. Due to the low number of children with obesity, the upper quartile of the BMI percentile was used as the cutoff point to define high body weight and used as the dichotomous variable in the ROC curve analysis. The ROC analysis demonstrated that NHR (AUC = 0.70; 95 % CI: 0.64–0.75; p < 0.01) and NLR (AUC = 0.63; 95 % CI: 0.55–0.71; p < 0.01) were the only markers with statistically significant Areas Under the Curve (AUC) for identifying high body weight (Fig. 2). Furthermore, NHR demonstrated a higher discriminative ability, with greater sensitivity and specificity surpassing all other markers, including NLR and HDL-C, on its own (Table 4); however, this difference was not statistically significant (DeLong test, p = 0.61).

**Table 4:** Area under the Roc curve.

Test Result Variable(s)	Area	Std. Error^a^	Asymptotic Sig.^b^	Asymptotic 95 % Confidence Interval
				Lower-Upper Bound
NHR	0.70	0.04	<0.01	0.64–0.75
NLR	0.63	0.06	0.02	0.55–0.71
MHR	0.54	0.06	0.56	0.41–0.66
TyG	0.51	0.06	0.87	0.39–0.63
TG-HDL-C	0.55	0.06	0.44	0.43–0.67
HDL-C	0.57	0.06	0.26	0.48–0.65

Dichotomous variable: High Body Weight defined by the upper BMI percentile Quartile.

The test result variable(s): TyG, TG-HDL-C, HDL-C has at least one tie between the positive actual state group and the negative actual state group.

a Under the nonparametric assumption.

b Null hypothesis: true area = 0.5.

In contrast, the ROC curves for TG-HDL-C, TyG, and HDL-C showed significant differences; but their AUC were of no significance and their values were lower than those of NHR and NLR. The optimal cutoff point for NHR was 1.27, with a sensitivity of 70 % and a specificity of 65 %. For NLR, the cutoff point was 1.69, with a sensitivity of 50 % and a specificity of 75 %.

### 4.6 Multiple linear regression analyses

Separate multiple linear regression models demonstrated that both NHR (coefficient = 0.24) and NLR (coefficient = 0.18), as dependent variables, were significantly associated with BMI, after adjusting for age, sex, blood pressure, and location.

## 5 Discussion

The risk associated with BMI as a marker of cardiometabolic diseases starts to accumulate significantly as early as childhood [4]. Combining different inflammatory biomarkers with conventional markers could improve risk prediction, but their significance in Indigenous children remains uncertain due to varying internal mechanisms. This study examined novel parameters, including NHR, NLR, MHR, TG-HDL-C, and TyG, in relation to high body weight among Indigenous Argentinean children living at different altitudes. ROC curve analysis revealed that NHR tended to perform better with a significant AUC for identifying high body weight, outperforming all other indices, including NLR and HDL-C alone. These findings suggest that NHR is strongly associated with high body weight and may serve as a reliable and cost-effective marker of cardiometabolic risk in Indigenous children from three low-income communities. Although our results highlight the potential utility of NHR as an early biomarker of cardiometabolic risk in Argentinean Indigenous children, it should be noted that NHR has not yet been widely validated in pediatric cohorts. Therefore, further prospective research should be performed to validate our results.

Prolonged and continuous exposure to obesity was linked to more adverse cardiometabolic outcomes in adults [14,15]. Furthermore, children with a lower socioeconomic level, as indicated by parental education, were linked to a higher likelihood of progressing to high BMI trajectory patterns in adulthood [16].

Despite the low prevalence of obesity (3.7 %) among these Indigenous children, their widespread low socioeconomic status—reflected by the fact that 76.3 % of parents had only elementary education or less—contributes to their increased vulnerability. Therefore, there is a need for affordable and simple laboratory markers to help identify children who already present associated cardiometabolic risk factors.

To date, no studies have explored the association between various inflammatory markers, dyslipidemia, and insulin resistance-related ratios and BMI in Indigenous schoolchildren living at different altitudes. Even though BMI percentiles are widely used in pediatric populations, altitude-related physiological adaptations may influence both metabolic and inflammatory markers [17]. In high-altitude settings, hypoxia can affect hemoglobin lipids and white cell counts through mechanisms involving increased erythropoiesis due to hypoxemia, oxidative stress, and systemic inflammation [17]. Accordingly, we showed that hemoglobin and hematocrit were significantly increased as altitude increased. In contrast, leukocyte counts, and neutrophil percentages were higher in Pampa del Indio (at sea level), possibly reflecting endemic parasitic infections (including Chagas disease), as indicated by the elevated eosinophil levels. Therefore, this could influence the relationship between hematological indices and cardiometabolic risk [17]. However, in our study, when we performed a multiple regression including location as an adjustment variable, no significant association was observed, suggesting that, within our data, altitude did not appear to influence these relationships. Future studies should nevertheless incorporate stratification or sensitivity analyses to further clarify the role of environmental context in shaping cardiometabolic profiles.

Most existing studies have focused on Western populations, with mixed results attributed to differences in study populations [18,19]. Investigating healthy Indigenous children from low socio-economic backgrounds without apparent comorbidities can help minimize the influence of confounding variables [[8], [9], [10]]. Identifying high-risk populations earlier in life is essential for the timely diagnosis of cardiometabolic diseases. Although combining multiple markers has shown promise in improving diagnostic and prognostic accuracy, the optimal combinations for early diagnosis and prognosis have yet to be determined [5]. Chronic inflammation and dyslipidemia are associated with obesity and, therefore, well-established risk factors for atherosclerosis and cardiometabolic diseases [[20], [21], [22], [23], [24]]. The continuous secretion of cytokines and adipokines from adipose tissue initiates an inflammatory cascade [20,21] involving various immune-related cells, including neutrophils, lymphocytes, monocytes, and macrophages [23,22]. In recent years, growing evidence has highlighted peripheral blood cell-derived biomarkers—such as the NLR, MHR, and NHR—as useful alternatives to traditional inflammatory markers like IL-6, CRP, and adiponectin [24]. These biomarkers have shown potential in detecting and predicting the presence and severity of systemic inflammation, particularly in relation to cardiometabolic diseases [25]. Environmental factors, including obesity, can lead to oxidative modification of HDL-C, resulting in dysfunctional HDL-C that lacks anti-atherosclerotic properties [6].

On the other hand, neutrophils contribute to increased peripheral insulin resistance by promoting inflammation in adipose tissue. Neutrophil-related abnormalities include neutrophilia and increased levels of reactive oxygen species [18,19]. However, Turkkan et al. [24] reported that inflammatory hematological indices in children with obesity were similar to those observed in normal-weight individuals. Similarly, Mărginean et al. [26] found no significant differences in NLR values between children and adolescents with obesity and their normal-weight counterparts. In contrast, Aydin et al. [27] identified a significant elevation in these indices among obese adolescents compared to healthy controls. In comparison, NHR was linked to cardiometabolic diseases and may serve as a predictor of adverse cardiovascular outcomes in individuals with prediabetes in China [19]. However, our study focuses on Indigenous schoolchildren and examines obesity-related outcomes, finding an AUC of 0.70 for NHR in identifying high BMI. These highlights both differences and potential universality: while Liu et al. [19] demonstrate the utility of NHR for long-term cardiovascular risk stratification in adults, our findings extend the relevance of NHR to pediatric obesity risk assessment in indigenous Argentinean children from socioeconomically deprived areas.

Moreover, NHR has been associated with Non-Alcoholic Fatty Liver Disease in healthy populations and has demonstrated superior prognostic value in elderly patients with heart attack [28,29]. Consistently, the current study found a significant relationship between NHR and NLR inflammatory indices and high body weight in Indigenous school-aged children. These discrepancies may be attributed to differences in study populations, including sample size, age range, severity of obesity, and gender distribution. ROC curves were generated in the present study to determine which marker had better discriminative performance than others for identifying children with high body weight.

We found that both NHR and NLR identify significantly high body weight, with no significant differences. However, NLR demonstrated a poor AUC curve (AUC <0.7), whereas NHR showed a good AUC. Although NHR AUC showed a better discriminative performance for identifying high body weight, this discrimination was moderate (AUC 0.70; sensitivity 70 %, specificity 65 %). Its utility should be interpreted with caution, as it does not imply complete clinical robustness. The present study found that the NHR AUC had better discriminative performance for identifying high body weight than all other indices, including NLR and HDL-C alone, in indigenous Argentinean children. The potential advantages of NHR may be explained by the anti-inflammatory effects of HDL-C, including the inhibition of neutrophil activation, as well as the central role of neutrophils in obesity-related inflammation. In addition, the higher sensitivity of NHR observed in Indigenous schoolchildren could be related to specific characteristics of lipid metabolism under conditions of low socioeconomic status, which may further support the biological plausibility of our findings.

These regions often face challenges related to the availability, accessibility, and affordability of healthcare resources [9,10,29]. NHR is a simple, inexpensive, non-invasive blood-based marker, and it may provide more valuable insights than single-parameter markers [19]. It could also be particularly valuable in resource-limited settings [19]. Therefore, this economic index will be a useful and cost-effective cardiometabolic risk assessment. However, if one can prevent obesity, one will ultimately prevent cardiometabolic disease [30]. With the growing rates of obesity among children, it is also important to establish healthy lifestyle habits at an early age [30]. It is important to highlight that this Indigenous child population does not yet exhibit a high prevalence of obesity. However, the rates of overweight and obesity were significantly higher in Pampa del Indio. If lifestyle changes occur, similar to those in Pampa del Indio—where residents rely exclusively on welfare and do not work—this could lead to a higher-risk population than other communities with greater resources. Given that this marker is both easy to measure and cost-effective, it could be highly valuable in these low-income Indigenous communities for assessing cardiometabolic risk.

*Community Engagement:* Our group has been conducting investigations with Indigenous communities in the Andean foothills of Salta for over 12 years. Later on, we established a connection with the provincial Ministry of Health, which has continued this work. In contrast, last year we visited Pampa del Indio (Chaco) for the first time, where we collaborated with the Universidad Nacional del Nordeste. Students and local nurses formed a group to continue providing health assistance, thereby strengthening sustainability and community empowerment.

## 6 Strength and limitations

This study presents several strengths over previous research. Firstly, it identifies an association between NHR and high body weight in a large school-age population-based sample of apparently normal Indigenous children. Additionally, these markers are cost-effective, non-invasive, easy to administer, and widely accessible. The high participation rate of the school children, the collection of fasting blood samples, and the use of ROC curve analysis to identify the most effective index for detecting high body weight are notable strengths of the study. Moreover, the study was conducted among school children from various Indigenous communities, making it a multicenter study. Finally, few, if any, previous studies on Indigenous school children have examined the association between these indices and high body weight.

Nevertheless, there are some limitations to consider. As a cross-sectional study, it cannot establish a causal relationship. Furthermore, potential confounding factors, such as family history of cardiometabolic diseases, physical activity, dietary intake, and pubertal status, were not evaluated in this population. Future longitudinal studies should incorporate lifestyle behaviors to strengthen the continuity of this research. Additionally, the moderate predictive capacity of NHR may limit its usefulness as a standalone marker in clinical practice. The findings of this study cannot be generalized to other populations, as the results were obtained from an Argentinean indigenous population with low socioeconomic backgrounds.

## 7 Conclusions

In the Argentine Indigenous schoolchildren, NHR demonstrated better discriminative performance compared with other markers for identifying high body weight. This suggests that NHR could serve as a reliable marker of cardiometabolic risk in Indigenous children from three resource-limited communities. As a simple, cost-effective, and non-invasive blood-based marker, NHR could be especially valuable in resource-limited settings for assessing cardiometabolic risk. Moreover, preventing obesity is crucial to reducing the burden of cardiometabolic diseases. Further research should be conducted in children from diverse ethnic and socioeconomic backgrounds to confirm these findings.

## CRediT authorship contribution statement

**Valeria Hirschler:** Writing – review & editing, Writing – original draft, Project administration, Methodology, Investigation, Formal analysis, Data curation, Conceptualization. **Silvia Lapertosa:** Supervision, Resources, Methodology. **Luis Antonio Castaño:** Resources, Conceptualization. **Gustavo Maccallini:** Software, Resources. **Claudio Gonzalez:** Writing – original draft, Methodology, Conceptualization.

## Funding

No funding was received for the conduct of the whole study.

## Declaration of competing interest

The authors declare that they have no known competing interests or personal relationships that could have appeared to influence the work reported in this paper.

## Data Availability

Data will be made available on request.
